# The Impact of Acoustic Wave Therapy on Viability and Differentiation Capacity of Human Adipose Stem Cells

**DOI:** 10.1111/jocd.70142

**Published:** 2025-03-27

**Authors:** Sheila Veronese, Riccardo Ossanna, Sara Ghazanfar Tehrani, Paolo Bernardi, Sima Khabouri, Velia Cannone, Maria Maddalena Nicoletti, Mario Goisis, Andrea Sbarbati

**Affiliations:** ^1^ Department of Neuroscience, Biomedicine, and Movement, Section of Anatomy and Histology University of Verona Verona Italy; ^2^ De Clinic Milan Italy; ^3^ Department of Precision Medicine University of Campania “Luigi Vanvitelli” Naples Italy

**Keywords:** adipose tissue, ADSCs, differentiation, extracellular vesicles, fat grafting, shock waves

## Abstract

**Background:**

Acoustic Wave Therapy (AWT) is a particular shock wave treatment that combines focused and radial shock waves, resulting in particular efficiency in the body's superficial layers. Its application before fat grafting has the potential to enhance it, favoring a better result of the graft.

**Aims:**

The aim of this study was to analyze the effects of AWT on fat tissue.

**Methods:**

Histological analysis of fat harvested from the abdomen of 40 subjects was evaluated. Twenty subjects had been pre‐treated with AWT, while 20 had not. Extraction and characterization of adipose‐derived stem cells (ADSCs) were performed for all. The multilineage differentiation capacity of extracted ADSCs and exosome extracellular vesicles' production from the fat samples were analyzed in both groups of specimens.

**Results:**

All 40 specimens presented both preservation of the structure of the tissue and of the cells, particularly of adipocytes. The cell growth resulted higher for pre‐treated samples. All ADSCs from all the samples were able to differentiate along adipogenic, chondrogenic, and osteogenic lineages. In particular, for pre‐treated samples, in the adipogenic lineage, a more advanced maturation phase was observed, and in the chondrogenic lineage, a chondroid matrix was evident around chondrocyte aggregates. All the samples reacted with exosome extracellular vesicles' production to stress.

**Conclusions:**

The results highlighted that AWT aligns with the in‐force minimal manipulation regulations, as the tissue and cells' structure and functionality were preserved. The types of differentiation observed permitted us to speculate about possible new applications in aesthetic and regenerative medicine.

## 1 Introduction

In 1980, Christian Chaussy successfully applied an extracorporeal acoustic wave at high pressure (> 100 MPa) for lithotripsy (ESWL, extracorporeal shock wave lithotripsy) [1]. From there, this revolutionary approach evolved into extracorporeal shock wave therapy (ESWT).

ESWT consists of the focalization of the energy derived from multiple acoustic waves in the area to treat. These waves have a high‐pressure amplitude (10–100 MPa), short duration (about 0.2 μs) and extremely rapid rise time (10–100 ns). In general, ESWT applies lower pressure with respect to ESWL, as it is applied to softer tissues to reduce pain, restore tissue functionality, and promote regeneration. Considering that when a propagating wave meets a tissue with a different density, it is partially reflected, and a certain amount of energy spreads in the surrounding tissues. This involves a deformation of the cell membranes with alteration of the intermolecular bonds [1, 2]. This aspect, initially considered secondary, has been re‐evaluated with time. The interaction of the waves with the tissues and cells has been investigated. It was discovered that it results in mechanotransduction, which targets particular cells, molecules, and tissues [3].

In the 1990s, the radial shock waves (RSW) were introduced. From the physical point of view, they are extracorporeal pressure waves and not extracorporeal shock waves, as the pressures used are 100 times inferior (0.1–1 MPa), and their length is 1000 times longer than those of ESWT [4]. They induce a strong tissue reaction at the application point and fade, deepening into the tissues due to the tissue density and fan diffusion. They do not focalize.

Radial waves permit taking advantage of the beneficial effects detected with shock waves in superficial tissues (≤ 50 mm of depth compared to the 200 mm of shock waves). Acoustic Wave Therapy (AWT) has been developed from these considerations, mainly for aesthetic and regenerative medicine. AWT combines RSW and ESWT. It has already been successfully used in different medical fields: orthopedic pathology therapies and chronic tissue inflammation treatment [5], wound healing [6, 7], face contouring [8, 9], and cellulite treatment [10, 11, 12]. AWT has induced various biological effects in aesthetic therapies, such as microcirculation stimulation, increased metabolic processes, and improved connective tissue elasticity [11]. Of particular interest is the stimulation of stem cells induced by this therapy [8, 9]. Nowadays, the use of mesenchymal stem cells (MSCs) is spreading in regenerative and medical fields. Based on MSCs, innovative cell therapies are employed to restore cells lost in many human disorders and enhance the repair processes of various tissues. The most relevant characteristics of MSCs are their self‐renewal ability and growth capacity, which could be influenced by multiple stimuli, such as nutrients, growth factors, and/or various physical stimulations. Among the latter is included AWT [13]. Recently, small extracellular vesicles (sEVs) secreted by stem cells have attracted much attention due to their innumerable advantages and regenerative capabilities similar to those of their parental cells [14, 15].

Extracellular vesicles (EVs) are small, membrane‐enclosed structures released by cells into the extracellular environment. By size‐based classification, they include small EVs and medium/large EVs (m/lEVs). sEVs, typically < 200 nm in diameter, originate from the endosomal compartment, while m/lEVs (> 200 nm) bud directly from the plasma membrane. EVs carry a variety of cargo, such as proteins, lipids, mRNA, and microRNA, facilitating intercellular communication and influencing numerous physiological and pathological processes. They are vital in immune modulation, cancer metastasis, and neurodegenerative disease progression. Due to their function in disease mechanisms and their potential as biomarkers, EVs are increasingly investigated for diagnostic and therapeutic applications [16].

Adipose tissue is known to be rich in stem cells [13, 17], which are able to secrete EVs largely. A significant increase in EVs' production is reported with different forms of mechanical stimulation [18] and by applying electroporation protocols [19]. Adipose tissue is largely used in various medical applications for grafting due to the adipose‐derived stem cells (ADSCs) it contains [20, 21]. In this procedure, fat is harvested from a donor site (usually, abdomen and thigh), processed, and re‐injected in the site for treatment. Before re‐injection, adipose tissue was enzymatically processed. Still, since 2017, with the introduction of the new regulatory limitations on minimal manipulation tissue requirements [22], many devices and techniques have been implemented for fat processing [23]. An optimal processing technique or combination of techniques has not yet been identified.

AWT might be used as fat pre‐treatment before the harvesting phase of fat grafting, with the aim of optimizing the harvested tissue. This type of pre‐treatment has been performed only twice in two completely different clinical applications: the remodeling of saddle nose [24] and the vulvovaginal atrophy treatment [25]. In the first study [24], of 97 patients considered, 32 were pre‐treated with AWT in the donor area before fat harvesting. Then, all the patients underwent fat grafting to correct their saddle noses. After the grafting, the leftover fat was analyzed, denoting a higher cellular growth of ADSCs in the pre‐treated samples. Moreover, at 3 and 12 months of follow‐up, the patients' satisfaction levels resulted better if they received the pre‐treatment, meaning more stable results over time. In the second study [25], of the patients treated for vaginal atrophy, 16 underwent fat grafting with the AWT pre‐treatment of the donor area. The study demonstrated that the pre‐treatment improved the regenerative capacity of the stem cells, albeit this treatment did not affect the cellular yield. The vaginal atrophy treatment appeared promising. A complete biological analysis of the effects of AWT on adipose tissue that could justify the good results obtained in the previous studies has never been done. For this reason, the present study is the first that aims to evaluate these effects and the effects of AWT on ADSCs.

## 2 Materials and Methods

### 2.1 Patients

In 2023, 40 patients underwent face and neck grafting. All patients were females ages 23–56. They had no previous surgery at the harvesting site that could have involved fibrosis or the presence of atrophic tissue. Exclusion criteria were: oncological subjects, obese people, people with metabolic disorders, or smokers. At the surgical time, all subjects were not assuming drugs.

Before fat harvesting, half of the subjects underwent the Acoustic Wave Therapy—AWT group, while the other half were not subjected to any pre‐treatment—not AWT group. The division of patients into the 2 groups was purely random. It was only avoided having heterogeneous groups by age. Applying this criterion, the two groups of patients were defined through computer‐generated lists. After fat harvesting, for half of the patients in the AWT group and half of the patients in the not AWT group, the fat was processed with a mechanical system. Subsequently, it was reinjected in the recipient site (face and neck).

The leftover fat of both groups was collected for morphological and functional characterization. The only data available concerning these specimens were related to their pre‐treatment with AWT and the type of processing they underwent.

All the procedures were conducted in full compliance with the ethical norms and standards of the Helsinki Declaration of 1975, as revised in 1983. Ethical review and approval were waived for this study because the fat samples analyzed were leftover anonymized material from the face and neck grafting procedures, commonly effectuated. Patients had been informed and agreed to the use of their leftover material for laboratory analysis.

### 2.2 Pre‐Treatment With AWT

AWT was performed by combining focused and radial acoustic waves, starting 3 weeks before fat harvesting. Detailing, the application was performed in a 10 × 15 cm area of the abdomen, corresponding to the harvesting area.

Before AWT, as its complement, vibration therapy was performed using the DUO‐LITH SD1 system and the V‐ACTOR HF V40 handpiece (Storz Medical AG, Tägerwilen, Switzerland). 3000 pulses of 3 bars were applied with a frequency of 50 Hz.

The focused shock waves were applied by the DUOLITH SD1 system and the C‐ACTOR C40 handpiece (Storz Medical AG, Tägerwilen, Switzerland). The amount of constant energy applied was 0.5 mJ/mm^2^ to promote a superficial action (≤ 15 mm in depth). A total of 2000 pulses were erogated at a frequency of 8 Hz.

Even the radial shock waves were applied by the DUOLITH SD1 system. The handpiece was the D‐ACTOR D20 (Storz Medical AG, Tägerwilen, Switzerland). Pressure waves were of 2.5 bar, and 4000 pulses were erogated with a frequency of 21 Hz.

AWT and the complementary therapy were repeated 6 times, twice a week, before fat harvesting, on the basis of a protocol defined by the manufacturer. After the treatment, there were no specific procedures for the patients, such as massages or topical cosmetics. Sun exposition was not avoided.

### 2.3 Fat Harvesting

Before harvesting, the area was infiltrated with Klein's solution, which was prepared by mixing 250 mL saline solution, 0.25 mL adrenaline 1 mg/mL and 10 mL lidocaine 2%. 150–200 mL of Klein's solution was prepared to harvest 60 mL of lipoaspirate.

Klein's solution induces local anesthesia and temporary vasoconstriction. It reduces bleeding and permits tumescence formation, which is necessary for the harvesting phase.

In the harvesting area, 2 mL of lidocaine 2% was infiltrated, and a small incision was performed to favor the cannulas' entrance. Through this, 120 mL of Klein's solution was infiltrated by a 16G cannula. After 7–8 min, the fat harvesting was performed by an 18G cannula (Go Easy system, Milan, Italy).

### 2.4 Fat Processing

Different mechanical processing systems were applied to half of the harvested fat (50% pre‐treated with AWT and 50% not pre‐treated): Goeasy.bio system Dermgraft (Go Easy system, Milan, Italy), Goeasy.bio enriched Nanograft (Go Easy system, Milan, Italy), Microfat (Lipocube, London, UK), Nanofat (Lipocube, London, UK), the Coleman technique, and the Seffi (Superficial Enhanced Fluid Fat Injection, Bologna, Italy). All systems met the minimal manipulation requirements. The number of samples treated by each system was 2–3 (no one was used predominantly concerning the other systems). To standardize the samples, samples from patients of different ages were considered for each system (2–3 patients in 2–3 different decades). In this way, for every system, the samples were obtained from the processing of fat harvested both from the youngest and oldest patients. The choice of which system to use was linked to the instrumentation available in the various centers where the grafting was performed. All the procedures were performed by a single surgeon operating in these different centers. To maintain consistency across the centers, the samples were progressively enumerated using a single database. Considering the limited applications, a specific analysis related to each processing system was not performed because it was not significant.

After processing, the required fat was reinjected, and the leftover processed fat (non‐reinjected fat) was collected in a sterilized plastic canister. Then, it was sent in a box filled with dry ice for characterization.

### 2.5 Histological Analysis of Fat Tissue Samples

After being fixed with paraformaldehyde 4% (Boster Biological Technology Co. Ltd) for 20 min, adipose tissue samples were washed with PBS 1X, dried, and embedded with Optimal Cutting Temperature compound (OCT). Samples were cryo‐sectioned in 14 μm‐thick transversal slices. Then, slides were dried under flow for 30 min and stored at −20°C for the subsequent histological analysis.

To evaluate the morphology of the fat tissue samples, slides were rehydrated with distilled water, stained with Hematoxylin (Sigma‐Aldrich, Milan, Italy) and counter‐stained with 1/10 Eosin (Sigma‐Aldrich, Milan, Italy). Slides were then dehydrated with increasing alcohol concentration (80%–95%–100% for 5 min each, and xylene twice for 5 min). Finally, a drop of mounting medium (Entellan) was added, and the slides were covered with the cover slip.

All slides were examined under an Olympus BX‐51 microscope (Olympus, Tokyo, Japan) equipped with a digital camera (DKY‐F58 CCD JVC, Yokohama, Japan).

### 2.6 Enzymatic Digestion for ADSCs Extraction

For each patient, 10 mL of lipoaspirate was digested with collagenase type I at a concentration of 1 mg/mL (GIBCO Life Technology, Monza, Italy). It was dissolved in Hank's Balanced Salt Solution (HBSS, GIBCO Life Technology, Monza, Italy) with 2% of Bovine Se‐rum Albumin (BSA, GIBCO Life Technology, Monza, Italy) for 45 min at 37°C. Complete culture medium (Dulbecco's Modified Eagle's Medium (DMEM), Sigma‐Aldrich, Italy), supplemented with 10% Fetal Bovine Serum (FBS, GIBCO Life Technologies, USA), 1% 1:1 Penicillin/Streptomycin (P/S solution, GIBCO Life Technologies, USA) and 0.6% Amphotericin B (GIBCO Life Technologies, USA), was added to neutralize the enzyme action. After neutralization, the samples were centrifuged at 3000 rpm for 5 min. The cell pellets were incubated with Erythrocyte Lysis Buffer 1X (Macs Miltenyi Biotec, Milan, Italy) for 10 min at room temperature. The cell suspensions were centrifuged and resuspended in 1 mL of complete culture medium. Finally, the cells were filtered through a 70 μm nylon mesh.

### 2.7 Cellular Yield

The number of extracted cells was calculated by dividing the number of extracted free cells by the fat processed volume to evaluate the cellular yield. The number of living cells was calculated using the Trypan Blue exclusion assay in a CytoSMART counter (Auto‐mated Image‐Based Cell Counter, version 1.5.0.16380, CytoSMART Technologies B.V., Eindhoven, Netherlands).

### 2.8 Cellular Growth Capacity

The extracted ADSCs were seeded on a 25 cm^2^ T‐flask with a complete culture medium and incubated in a humidified atmosphere at 37°C with 5% CO_2_. The first medium change was performed after 72 h from the enzymatic digestion, and the subsequent changes were performed every 48 h. The cellular growth evaluation was obtained by counting the cells after 7, 14, and 21 days of culture. The obtained cell numbers were normalized by dividing them by the corresponding cellular yield.

### 2.9 Differentiation Potential

The differentiation potential was evaluated in vitro for the biological samples pre‐treated with AWT and those not treated (notAWT). Differentiation was performed employing the cultured cells at passage 2 of expansion. Three different types of differentiation were analyzed: adipogenic, chondrogenic, and osteogenic.

#### 2.9.1 Adipogenic Differentiation

To grow adherent cells, 5000 cells were seeded onto a 12‐well plate containing one glass slide per well. The cells were incubated at 37°C, 5% CO_2_ for 24 h. Then, the culture medium was replaced entirely by adipogenic media Basal Medium with SupplementMix of the Mesenchymal Stem Cell Adipogenic Differentiation Medium 2 kit (Sigma‐Aldrich, Milan, Italy). To evaluate the adipogenic differentiation capacity, after 7 and 14 days of incubation, the cells were fixed with Baker's fixative (Bio‐Optica, Milan, Italy) for 10 min at 4°C. Then, they were washed with tap water for 10 min, stained with Oil‐Red‐Oil solution (Bio‐Optica, Milan, Italy) for 10 min, and stained with Mayer's hematoxylin (Bio‐Optica, Milan, Italy) for 5 min. After these phases, the glass coverslips were mounted with Mount Quick aqueous (Bio‐Optica, Milan, Italy).

#### 2.9.2 Chondrogenic Differentiation

1 × 10^6^ cells resuspended in 5 μL of complete culture media were seeded in a 12‐well plate. After 2 h, the chondrogenic media StemPro Osteocyte/Chondrocyte Differentiation Basal Medium with the StemPro Chondrogenesis Supplement of the StemPro Chondrogenesis Differentiation Kit (GIBCO Life Technology, Monza, Italy) was added. This media was changed every 3 days. After 1 and 14 days of incubation, cells were fixed with 4% formaldehyde (Bioptica, Milan, Italy) in Phosphate‐Buffered Saline (PBS 0.05 M) for 30 min at 4°C. Then they were washed twice with distilled water and stained with Alcian Blue solution (Merck KGaA, Darmstadt, Germany) for 40 min and with Nuclear Fast Red (Bioptica, Milan, Italy) for 20 min. After a brief dehydration, the glass coverslips were mounted with Entellan (Merck KGaA, Darmstadt, Germany).

#### 2.9.3 Osteogenic Differentiation

Five thousand cells were seeded on a 12‐well plate with complete culture media. After 24 h, the media was replaced with the osteogenic media StemPro Osteocyte/Chondrocyte Differentiation Basal Medium with StemPro Osteogenesis Supplement of the StemPro Osteogenesis Differentiation Kit (GIBCO Life Technology, Monza, Italy). To evaluate the osteogenic differentiation capacity, after 7 and 14 days of incubation, the cells were fixed with 4% formaldehyde (Bioptica, Milan, Italy) in PBS 0.05 M for 30 min at 4°C. Then, they were washed twice with distilled water and incubated with Alizarin Red Solution (Merck KGaA, Darmstadt, Germany) for 2–3 min and Mayer's hematoxylin (Bio‐Optica, Milan, Italy) for 30 s. Finally, after a brief dehydration, the glass coverslips were mounted with Entellan (Merck KGaA, Darmstadt, Germany).

#### 2.9.4 Imaging

Images of stained cells were obtained using a bright‐field optical microscope, Olympus BX‐51 (Olympus, Tokyo, Japan), equipped with a digital camera (DKY‐F58 CCD JVC, Yokohama, Japan). The slides were gently cleaned with ethanol and placed on the microscope slide holder. For each slide, 15 images were acquired. A 20× objective was used for quantifying lipid droplets, while a 10× objective was used for quantifying calcified and collagen matrices. To standardize quantification, all the acquired images contained 8–12 cells.

#### 2.9.5 Analysis

Semiquantitative analysis was performed using ImageJ.JS software, version v0.5.6 (National Institute of Mental Health, Bethesda, Maryland, USA). Samples from 3 control patients and 3 patients pre‐treated with AWT were considered for each differentiation test. The differentiation tests were conducted in triplicate for all the patients. For each replica, 15 images were captured by a 10× objective according to a systematic randomized protocol, and their entire area was considered a Region of Interest (ROI). As red spots coincide with lipid droplets, their number on the cells' cytoplasm was counted to evaluate adipogenic differentiation in these ROIs. The number of blue spots on the cells' cytoplasm was counted to assess chondrogenic differentiation, as these spots coincide with cartilage fragments.

Differentiation capability was assessed by calculating the mean area of lipid droplets for adipogenic differentiation, the mean area of collagen aggregates (stained in blue) for chondrogenic differentiation, and the mean area of calcification deposits (stained in red) for osteogenic differentiation.

### 2.10 Extracellular Vesicles

The EVs' production of cell samples was evaluated from the supernatant obtained during the cell culture. Briefly, MSCs were growth in complete culture medium (Dulbecco's Modified Eagle's Medium (DMEM), Sigma‐Aldrich, Italy), supplemented with 10% Fetal Bovine Serum (FBS, GIBCO Life Technologies, USA), and 1% 1:1 Penicillin/Streptomycin (P/S solution, GIBCO Life Technologies, USA). After 9 days of culture, FBS was removed from the cell culture medium to avoid the contamination of the vesicles from the serum. After a further 2 days, the supernatants of cells were collected and then centrifuged at 3500 rpm for 7 min. Finally, the supernatant parts were filtered through a 0.22 μm nylon mesh to remove cell debris. According to manufacturer instructions, EVs were extracted using the PureExo Exosome Isolation Kit (101Bio, Mountain View, CA, USA).

The size and concentrations of the EVs were measured by the Nanoparticles Tracking Analysis System (Nanosight NS300). All samples were diluted in PBS to obtain a final volume of 800 μL to allow the measurements. For each measurement, three 1‐min videos were captured under the following conditions: 25°C cell temperature, 20 μL/s syringe speed, and 488 nm laser wavelength. The acquisition parameters were set according to the manufacturer's software (NanoSight NS300). The Nanoparticles Tracking Analysis 3.4 software (Malvern Panalytic) was used to acquire and analyze the videos.

### 2.11 Statistical Analysis

Statistical analyses were performed using GraphPad Prism, version 7.03 for Windows (GraphPad Software, La Jolla, CA, USA).

The cellular yield, the number of cells/mL, was expressed as mean ± standard error of the mean (SEM). A *t*‐student test was performed to evaluate cellular yield differences between AWT and not AWT samples, considering a significance level of 5% (*p*‐value < 0.05).

The cell growth was obtained by the number of cells normalized, that is, by dividing the raw experimental number by the corresponding number of cells in the yield. The cell growth was expressed as mean ± SEM. A two‐way ANOVA test was performed to compare the cell growth of the two groups of samples. A significance level of 5% (*p*‐value < 0.05) was considered.

For adipogenic differentiation, the full field 20× objective was considered a region of interest (ROI). The number of lipid droplets per ROI was expressed as mean ± SEM. The lipid droplet area, measured in μm^2^, was expressed as mean ± SEM.

For chondrogenic differentiation, the full field 20× objective was considered as ROI. The number of chondrocyte clusters per ROI was expressed as mean ± SEM. The mean area of these aggregated, measured in μm^2^, was expressed as mean ± SEM.

The calcium deposit area, measured in μm^2^, was expressed as mean ± SEM for osteogenic differentiation.

A *t*‐student test was used to perform a statistical comparison between all the types of differentiation capability of the cells of 2 groups of people. The values *p* ≤ 0.05, *p* ≤ 0.001, and *p* ≤ 0.0001 were assumed as significance levels.

EVs' dimensions were reported as mean ± SEM of three measurements.

### 2.12 Clinical Outcomes

The clinical evaluation of the fat graft survival, tissue integration, and patient outcomes was performed by the surgeon who treated the patients.

## 3 Results

### 3.1 Histological Analysis

**FIGURE 1:**
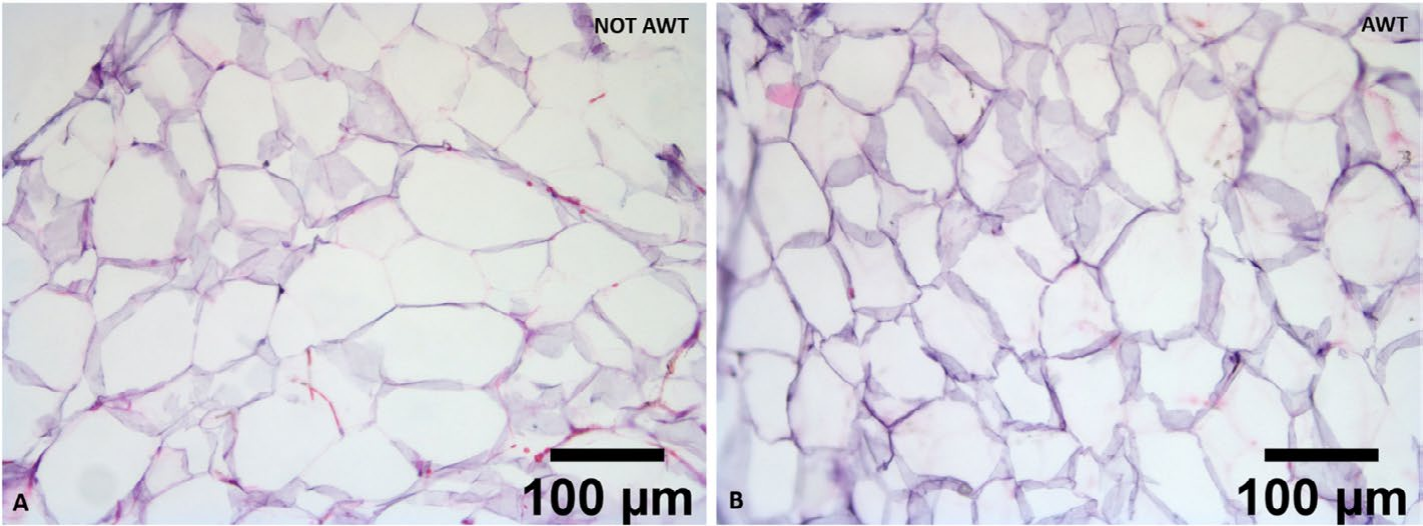
Histological analysis of fat harvested and processed with the Dermgraft system. (A) Sample harvested without previous in vivo fat processing and (B) sample harvested after 3 weeks of treatment with AWT. Adipocyte and ECM morphology appears preserved in both samples. The diameter of adipocytes results smaller for the samples pretreated with AWT.

The adipose tissue micro fragments obtained after fat harvesting of both pre‐treated with AWT and not pre‐treated (notAWT) fat presented a well‐defined and intact morphology when processed with Coleman's procedure and the Dermgraft system. The structure of the adipocytes appeared wholly preserved, with perfect integrity and stability. Moreover, they seemed to be in a healthy state. In addition, even the extra‐cellular matrix (ECM), which represents one of the most important components of fat‐derived products, appeared well‐structured and preserved. A slight reduction in adipocytes' diameters was observed in the AWT samples (Figure 1). Independently from the AWT pre‐treatment of the fat, the structure appeared partially preserved when the adipose tissue micro fragments were processed with the Microgrant and Seffi systems, according to the processing technique. The structure was completely not preserved when the fat was processed by the Nanofat and Nanograft systems.

### 3.2 Cellular Yield

Cellular yield was obtained from the total number of extracted ADSCs. The number of cells/mL in the samples pre‐treated with AWT was 5.7 × 10^5^ ± 1.47 × 10^5^ (on 17 samples), while the number of cells/mL in the samples not pre‐treated was 4.33 × 10^5^ ± 0.99 × 10^5^ (on 17 samples). No significant differences were detected between the experimental groups, even if a better trend was evident for the group of pre‐treated samples.

### 3.3 ADSCs Culture and Growth Capacity

**FIGURE 2:**
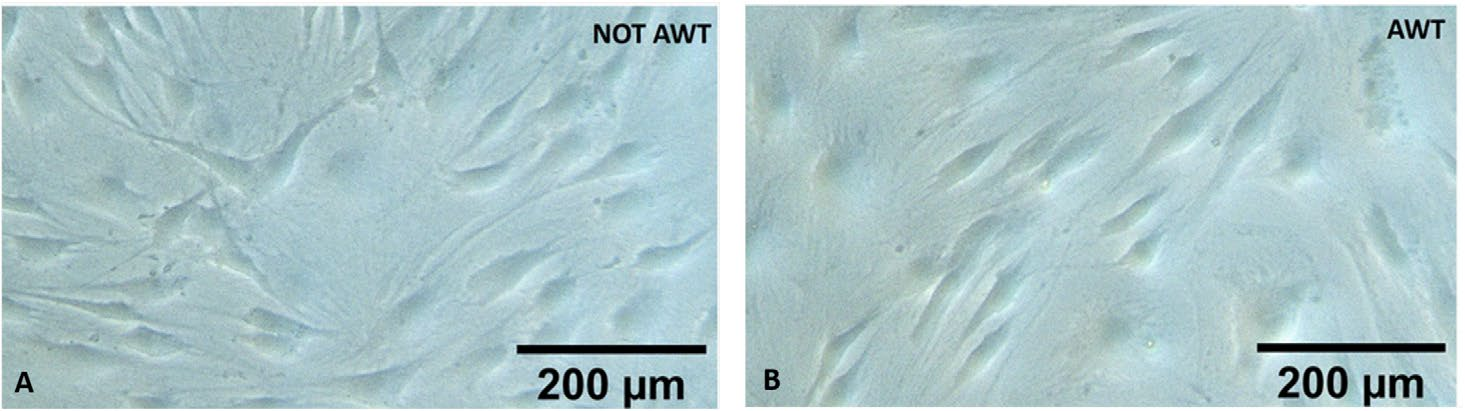
ADSCs culture. (A) ADSCs of the samples harvested without previous AWT pre‐treatment and (B) ADSCs of the samples harvested after 3 weeks of treatment with AWT. In both samples, ADSCs present a fibroblast‐like shape. They appear vital and without morphological alterations.

In both AWT and notAWT cases, the ADSCs culture presented cells well attached to the flask with the right dimension. It was also noted that all the cells had a fibroblast‐like and elongated shape, with branches. Moreover, no membrane permeability alterations were observed, and no apoptotic or necrotic cells were detected (Figure 2).

**FIGURE 3:**
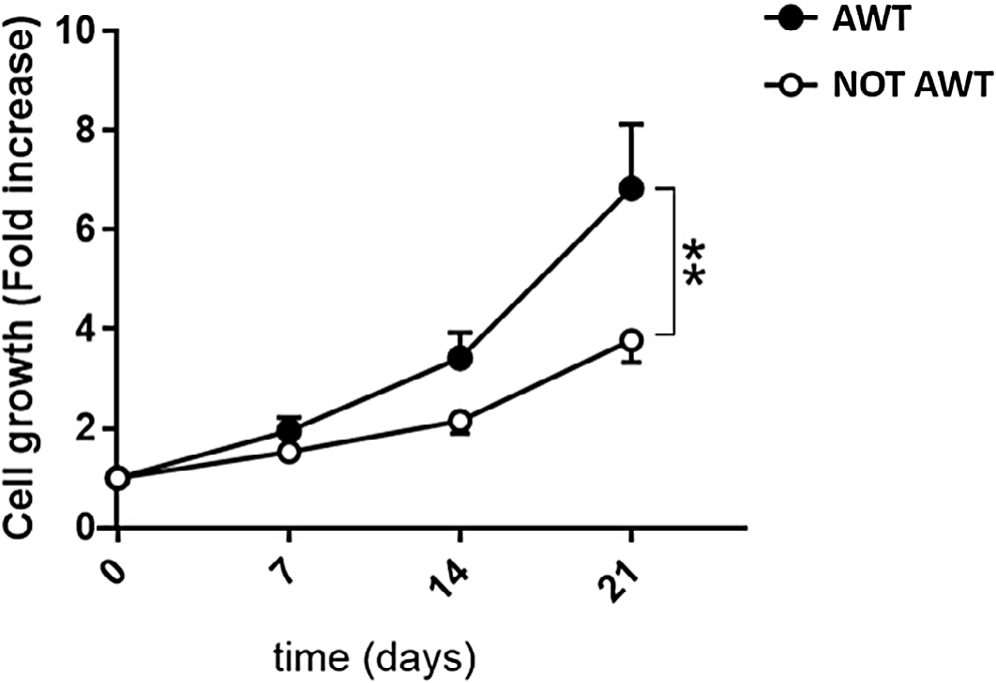
Cellular growth of both the sample types. Cell growth is expressed as the normalized number of cells. Normalization is performed by dividing the raw number of cells by the corresponding number of cells in the yield. The cellular growth is statistically different after 21 days of incubation. This demonstrates a higher proliferative ability of ADSCs obtained from the samples pre‐treated with AWT. **p ≤ 0.01.

Data on the cell growth ability of the ADSCs extracted from the pre‐treated with AWT and not pretreated fat highlighted a statistically higher ADSCs growth potential for the samples treated (Figure 3).

### 3.4 Multilineage Differentiation

**FIGURE 4:**
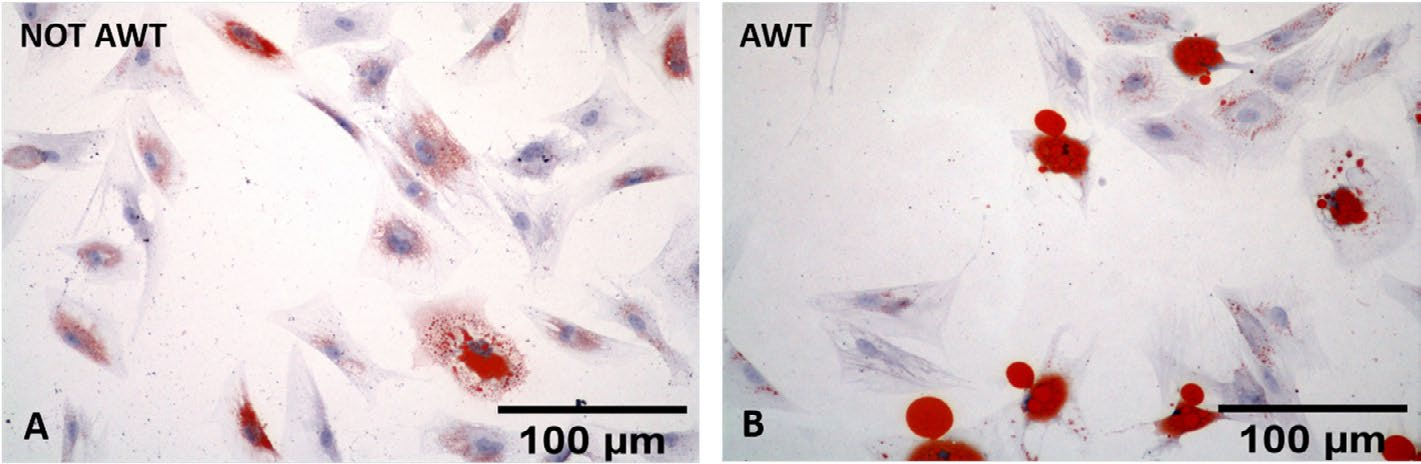
Adipogenic differentiation evaluated after 14 days of cell incubation for the cells of notAWT samples (A) and the cells of AWT samples (B). The cells obtained from the non‐treated samples appear polygonal or spindle‐shaped, yet to be confluent. In the cytoplasm, lipid accumulation appears characterized by finely dispersed droplets. The morphology of cells obtained from samples pre‐treated with AWT is similar to those obtained from the non‐treated samples. The lipid accumulation appears in large, rounded droplets surrounded by smaller droplets. This corresponds to a more advanced phase of maturation of adipocytes obtained from samples pre‐treated with AWT.

**FIGURE 5:**
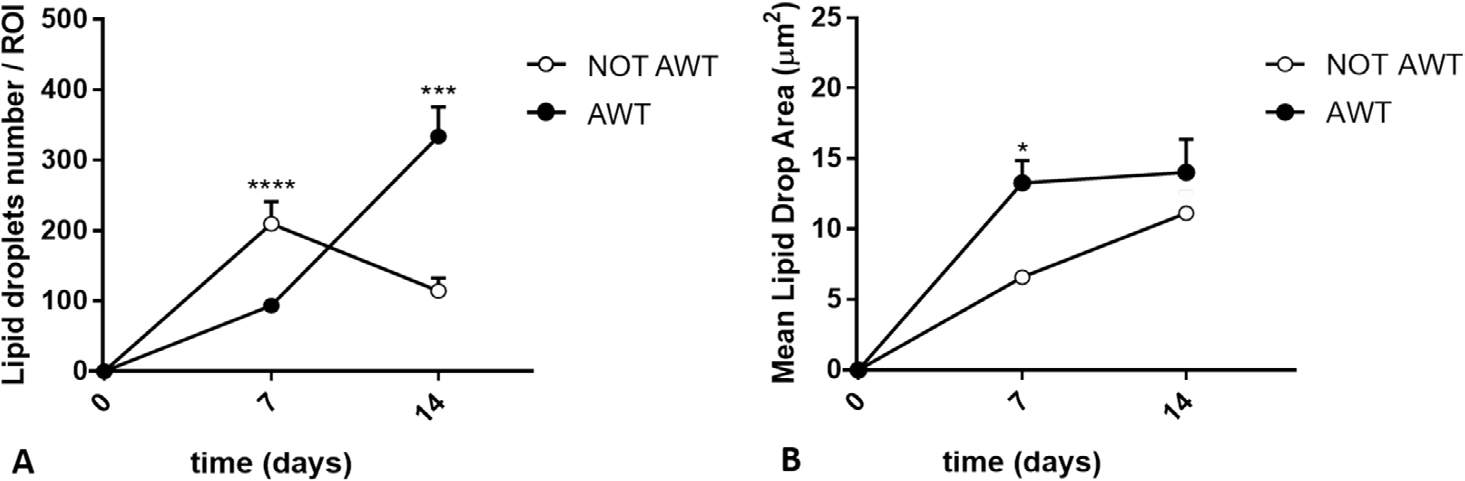
Representation of the number of lipid droplets per ROI and the mean area of lipid droplets. The ROI corresponds to the full field 20× objective. (A) Comparing the number of lipid droplets per ROI at 7 and 14 days after incubation between the notAWT and AWT samples, the statistical analysis highlights significant differences both at 7 and 14 days, with a prominent increase in the number of droplets at 14 days for the cells obtained from samples pre‐treated with AWT. (B) The dimension of the lipid droplets appears wider for the cells obtained from samples pre‐treated with AWT than that obtained from notAWT samples, already after 7 days of incubation. *p ≤ 0.05, ***p ≤ 0.001, ****p ≤ 0.0001.

Both samples from the notAWT and AWT people presented adipogenic differentiation capability (Figure 4). After 7 days of incubation, the mean amount of lipid droplets was higher for the notAWT cells concerning the AWT cells, but the average size of these droplets was larger for the AWT cells (Figure 5). After 14 days of incubation, the AWT cells presented more lipid droplets and a wider mean area (size of droplets) than notAWT cells.

**FIGURE 6:**
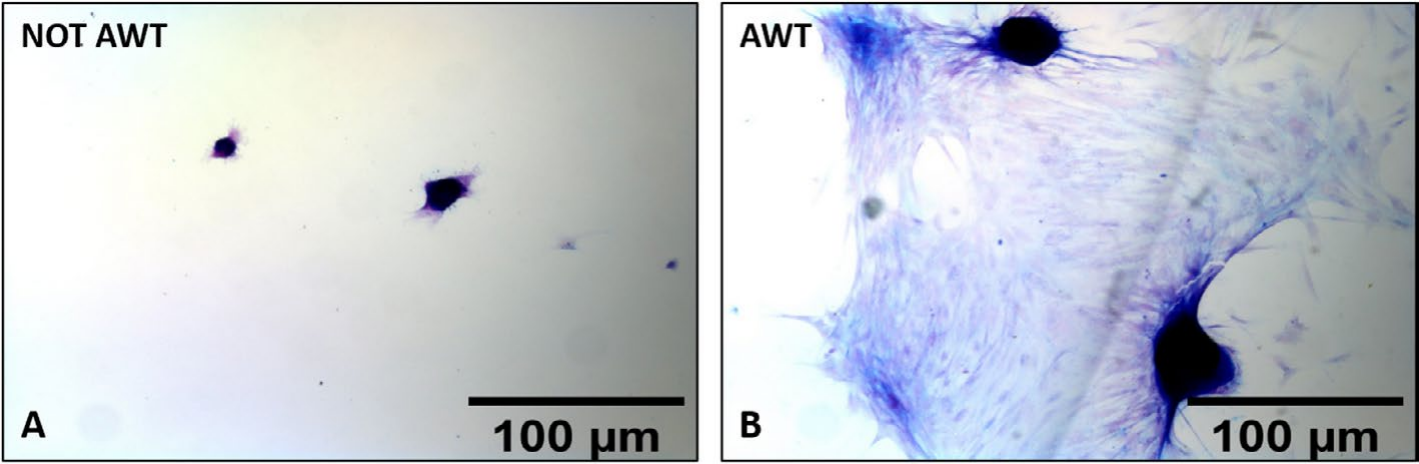
Chondrogenic differentiation evaluated after 14 days of cell incubation for the cells of notAWT samples (A) and the cells of AWT samples (B). Cells obtained from notAWT samples appear as not confluent round or polygonal elements, intensely colored. Cells obtained from samples pre‐treated with AWT present a similar morphology to that obtained from notAWT samples but broader in size. In these last samples, it is evident the presence of a chondroid matrix faintly colorable.

**FIGURE 7:**
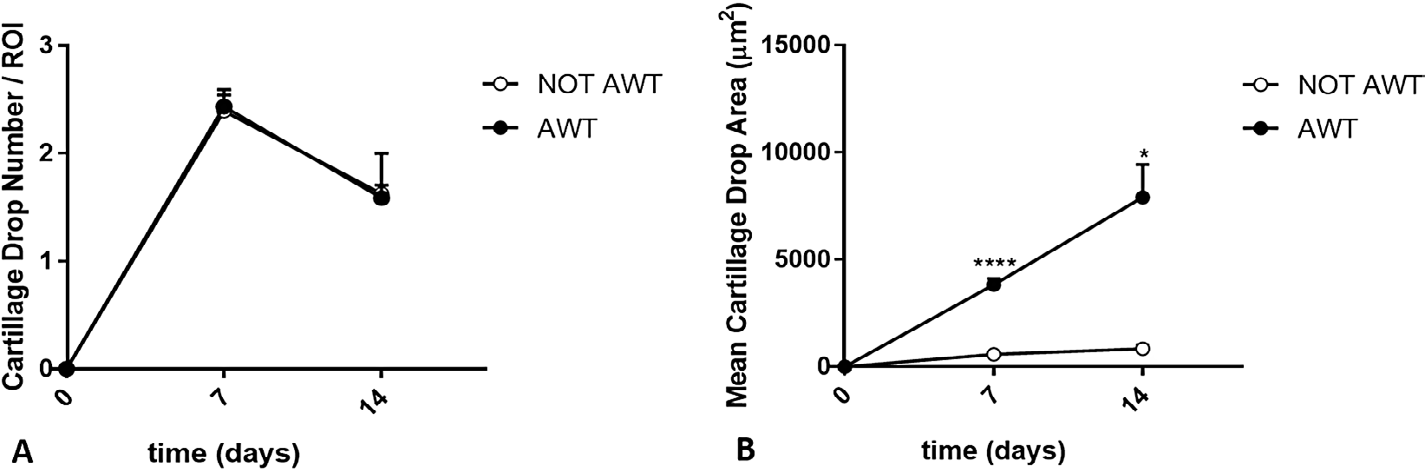
Representation of the number of chondrocyte clusters per ROI and the mean area of these aggregates. The ROI corresponds to the full field 20× objective. (A) The statistical analysis highlights no significant differences in the number of aggregates per ROI both at 7 and 14 days after incubation between the AWT and notAWT samples. (B) The dimension of the clusters appears statistically significantly wider for the cells obtained from samples pre‐treated with AWT than that obtained from notAWT samples, already after 7 days of incubation. *p ≤ 0.05, ****p ≤ 0.0001.

The data analysis related to chondrogenic differentiation highlighted that chondrocyte clusters were larger for samples pre‐treated with AWT than those obtained from not AWT samples (Figure 6). The number of fragments in the ROIs was not statistically different between the two groups of samples. In contrast, according to the morphological evaluation, the area of the aggregates obtained from samples pre‐treated with AWT was statistically wider than that obtained from not AWT samples (Figure 7).

**FIGURE 8:**
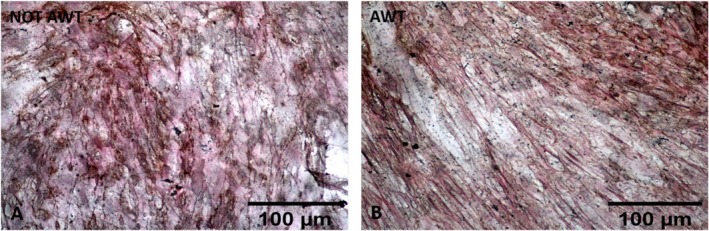
Osteogenic differentiation evaluated after 14 days of cell incubation for the cells of notAWT samples (A) and the cells of AWT samples (B). All the cells appear spindle‐shaped. They are immersed in a matrix rich in micro‐calcifications diffused in all the microscopic fields. Still, they are particularly abundant at the level of cell membranes, where they appear as rounded micro‐aggregates.

**FIGURE 9:**
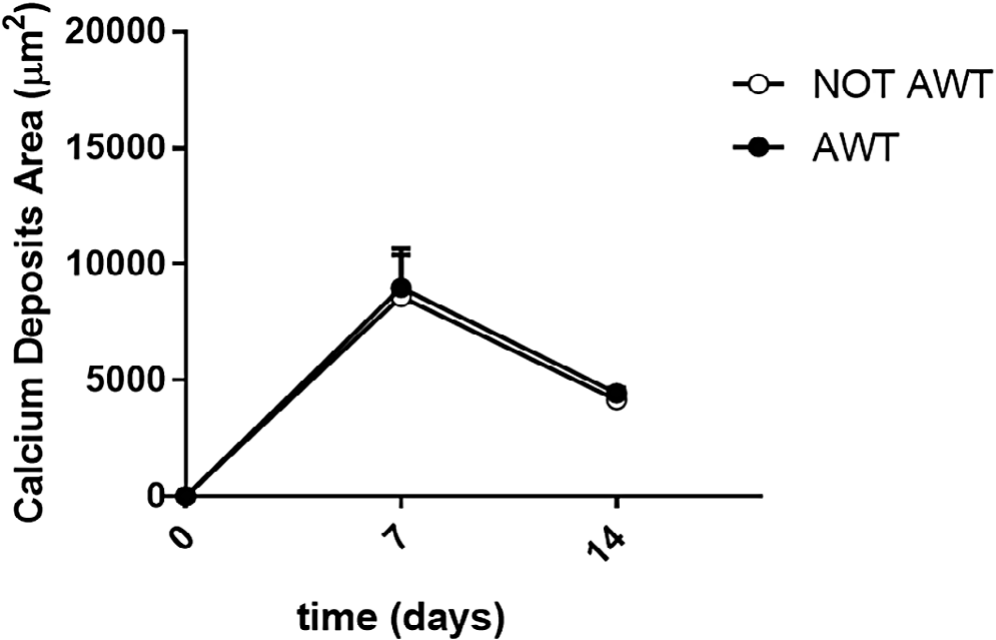
Representation of the mean area of calcium deposits. The statistical analysis highlights no significant differences in the dimensions of the calcium deposits between the cells obtained from notAWT samples and samples pre‐treated with AWT.

Regarding osteogenic differentiation, both samples from the notAWT and AWT patients presented calcium deposit areas. Morphologically, these areas appeared similar (Figure 8), even if a slight reduction in the number of cells seemed to be present in samples pre‐treated with AWT. The dimensions of the calcium deposits in the areas did not appear statistically different (Figure 9).

### 3.5 Extracellular Vesicles

Both samples from the notAWT and AWT people presented a high secretion in extracellular vesicles. The analysis did not highlight differences in both the mean size and the size mode of EVs (Table 1).

**TABLE 1:** Measurement of EVs' size expressed as mean and mode of EVs' diameter.

Group	Mean	Mode
notAWT	168.1 ± 4.5 nm*	101.6 ± 4.5 nm*
AWT	183.3 ± 6.2 nm*	131.2 ± 14.4 nm*

* *p* > 0.05 not statistically significant difference.

Concerning concentrations of EVs, they resulted slightly higher in the samples pre‐treated with AWT than in the not AWT samples (2.49 × 10^8^ particles/mL for AWT vs. 2.36 × 10^8^ particles/mL for notAWT).

### 3.6 Clinical Outcomes

The surgeon who treated the patients reported a good integration of both the fat pre‐treated with AWT and not pre‐treated, without any difference. He noted a higher survival rate of the fat pre‐treated with AWT than that of the not‐treated. This seems evident from the persistence of the grafting aesthetic results, which seem to have been maintained for more than 1 year.

## 4 Discussion

The combined application of focused and radial shock waves with reduced energy induces its effects superficially in the first 5–6 cm of depth. Therefore, the resulting therapy, named AWT, is extremely promising in all aesthetic fields, from cellulite [10, 11, 12, 26, 27] to contouring [26, 28, 29], face treatments [8, 9], and many other applications.

Moreover, the cell activation of superficial layers of the body correlated to this therapy could be a breakthrough in regenerative applications. Remarkably, considering that fat grafting is a widely diffused technique for multiple aesthetic and regenerative procedures [30], treating adipose tissue before fat harvesting could prepare it to better express its properties in the injection sites. One of the main limitations of fat grafting is that, after applying some procedures, the number of ADSCs present in the tissue to re‐inject is limited [31, 32]. Therefore, a system that is able to promote ADSCs proliferation without altering their properties and nature might be a breakthrough. AWT has the characteristics to be a similar technique. To verify this possibility, the fat harvested pre‐treated and not with AWT was characterized in this study, and its properties and stemness were evaluated.

The histological analysis performed in this study demonstrated that AWT does not influence the tissue and adipocytes' structure. The data related to the preservation of the ECM is fascinating. In fact, this component plays an essential role in cellular differentiation, migration, and survival [33, 34, 35, 36]. Furthermore, it has a role in tissue elasticity and stability because it composes the surrounding structure of any tissue in the body, and it gives mechanical support thanks to the adhesion with any cells [33, 34, 35]. The preservation of the structure of all the adipose tissue components harvested is the first key result, as it demonstrates that AWT performs a minimal manipulation of the cells, according to the in‐force regulations [22]. The slight reduction in adipocytes' diameters observed in the AWT samples might signify an activation of the adipocytes promoted by AWT. This action is not only crucial for the regenerative process of the injected fat but also justifies the use of AWT. In addition, it introduces the possibility of reducing time integrations between the donor fat and the receiving site.

The absolute number and the vitality of the extracted ADSCs, not different between the different samples (pre‐treated and not with AWT), highlighted phenotypic and kinetics preservation. The absence of membrane permeability alterations and apoptotic and necrotic cells indicates a healthy condition of ADSCs. This means that AWT resulted not to be a stressed procedure for the tissue. However, one of the most critical determinants of ADSCs' quality is their proliferation capacity. The statistically higher cell growth potential of ADSCs from AWT samples compared to non‐AWT ones demonstrated that AWT holds a greater proliferation capacity in fat‐derived stem cells. This is extremely impressive, considering that a mechanical processing system processed all the harvested fat before reinjection. The harvesting procedure and the subsequent processing phase may determine a modification in cell reactivity, slowing the integration in the receiving site. This study has a significant limitation in that it uses fat tissue processed with different types of devices. Nonetheless, in all the cases, independently of the device used, the fat treated before the harvesting phase presented cells with greater vitality and proliferative capacity. Consequently, the obtained results highlighted that AWT permits the maintenance of the ability of the cells to react to different procedures and adapt to new environments, favoring local tissue regeneration. The enhanced ADSC proliferation might be the solution for compensating the small number of ADSCs generally obtainable from mechanical fat processing systems. Usually, this number is smaller concerning enzymatic processing systems [31, 32], which are no longer usable in the surgical room. The high proliferation might imply a reduction in recovery time in regenerative applications and, in general, a more efficient action in the fat injection sites.

The complete differentiation capability, that is, the maintenance of a multilineage differentiation ability of ADSCs after AWT fat treatment, is also exciting.

In adipogenic differentiation, a difference in lipid droplet size in the cell cytoplasm was highlighted. The broader dimensions of the droplets of the cells of the samples pre‐treated with AWT might be explained by the confluence of smaller droplets in the larger ones, clearly observable in the histological specimens (Figure 4B). This natural behavior has already been demonstrated by Boschi et al. [37]. In addition, this aspect suggests a discrete metabolic stimulation degree in the re‐injection sites, which is higher for samples pre‐treated with AWT than for notAWT samples.

Concerning chondrogenic differentiation, the promotion of cartilage regeneration over time is of undoubted interest, mainly in the orthopedic field and regenerative medicine.

Results about osteogenic differentiation have to be carefully evaluated. The limited amount of data in this study does not permit inferring definite conclusions. Nevertheless, the AWT seems not only not to improve/promote osteogenic differentiation but to inhibit it. This suggests that its use be avoided when bone regeneration is required. Instead, in the case of ossifications, such as in periarticular ossifications after joint surgeries, the surgical resolution of the ossifications might be coupled with fat grafting, with fat pre‐treated with AWT. In this way, it could enhance healing, disfavouring new ossification processes. Fat grafting, with fat pre‐treated with AWT, might also be used as a periodic prevention treatment to avoid periarticular ossifications, improving and extending the results of limbs' prosthetic surgeries and, consequently, improving patients' quality of life.

Finally, the EVs' analysis permitted us to highlight how the AWT did not affect EVs' formation. This contributes to proof that AWT is a treatment that performs minimal manipulation of fat tissue [22]. The slightly higher concentration of EVs in samples pre‐treated with AWT than non‐AWT samples might signify that AWT has a role in modulating EVs' production. An in‐depth biological analysis of this modulation aspect and how this may be clinically used is undoubtedly to be performed.

This study offers many other exciting aspects to consider further. It could be extremely interesting to investigate the behavior of the cells after a combination of AWT and specific processing methodologies (decantation, washing,…) or with specific devices.

It could also be exciting to investigate the clinical effects correlated to the significant cell activity and how this may influence tissue healing/repair/regeneration or improve surgical results.

Moreover, applying the AWT in the donor sites could be extremely interesting in increasing the tissue reaction to the fat injection. In this case, the exact time in which the AWT should be performed should be investigated.

The main limitation of this study is the lack of clinical results. A short‐ and long‐term clinical assessment of the two groups should be performed, also considering the patients' satisfaction. Moreover, the effects of fat grafting should be evaluated, considering the correspondence with histological results. Clinical results, already present in the literature, suggested the pre‐treatment with AWT, which seemed to enhance results, warranting a higher percentage of tissue survival and making them more durable [24, 25]. However, the two studies were related to two completely different applications, and the most extended follow‐up evaluated was 1 year. The good integration noted by the surgeon and the fat survival rate, which are correlated to the aesthetic persistence of the results, seem to confirm the literature data. Undoubtedly, further studies have to be performed, both in vitro and, mainly, targeted clinical trials.

It should be added that the patients of this study presented a recovery time correlated to the processing system used. For instance, Coleman's procedure generally implies some days of recovery time, according to the authors' experience. In this study, all the patients whose fat was processed with Coleman's procedure presented this recovery time independently from the AWT pre‐treatment. Thus, recovery times do not seem to be influenced by the AWT treatment.

Another limitation of this study is the limited number of subjects considered and the fact that these were all females. Further research with different study groups is necessary.

In conclusion, the results obtained highlighted that AWT is a minimal manipulation technique. Its application as a pre‐treatment of fat, before the fat grafting procedure, permits the preservation of the fat, resulting in a high number of ADSCs. Therefore, adipogenic and chondrogenic differentiation are favored, and this could be a crucial aspect in several aesthetic, plastic, and regenerative procedures. Indeed, this could favor long‐term fat retention, reducing recovery times with easier tissue integration and regeneration due to the presence of numerous ADSCs. This could, in turn, reduce the onset of fibrosis and scarring. Improved integration could also reduce the need for secondary maintenance treatments.

Undoubtedly, the observed results open the perspective to numerous further possible applications and studies.

## Author Contributions

**Sheila Veronese:** methodology, formal analysis, data curation, writing – original draft preparation, writing – review and editing, project administration. **Riccardo Ossanna:** software, validation, formal analysis, investigation, data curation, writing – original draft preparation. **Sara Ghazanfar Tehrani:** software, validation, formal analysis, investigation, data curation, writing – original draft preparation. **Paolo Bernardi:** software, validation, formal analysis, investigation. **Sima Khabouri:** investigation. **Velia Cannone:** investigation. **Maria Maddalena Nicoletti:** writing – review and editing. **Mario Goisis:** conceptualization, methodology, investigation, writing – review and editing. **Andrea Sbarbati:** conceptualization, methodology, formal analysis, writing – review and editing, supervision, project administration.

## Ethics Statement

The study was conducted in accordance with the Declaration of Helsinki. Ethical review and approval were waived for this study because the fat samples analyzed were leftover material from face and neck grafting procedures, commonly effectuated.

## Consent

All the participants gave informed consent for the analysis and publication of data obtained from the analysis of their leftover material.

## Conflicts of Interest

The authors declare no conflicts of interest.

## Data Availability

The data that support the findings of this study are available from the corresponding author upon reasonable request.
